# Quantitative Shotgun Proteomic Analysis of Bacteria after Overexpression of Recombinant Spider Miniature Spidroin, MaSp1

**DOI:** 10.3390/ijms25063556

**Published:** 2024-03-21

**Authors:** Kathryn Randene, J Alexander Hoang Mendoza, Michael Ysit, Craig Vierra

**Affiliations:** 1 Department of Biological Sciences, University of the Pacific, Stockton, CA 95211, USA; k_randene@u.pacific.edu (K.R.); michael.ysit@gmail.com (M.Y.); 2 Japantown Dental, San Jose, CA 95112, USA

**Keywords:** spider silk, recombinant spidroin, black widow spider, proteomics, MaSp1, bacteria

## Abstract

Spider silk has extraordinary mechanical properties, displaying high tensile strength, elasticity, and toughness. Given the high performance of natural fibers, one of the long-term goals of the silk community is to manufacture large-scale synthetic spider silk. This process requires vast quantities of recombinant proteins for wet-spinning applications. Attempts to synthesize large amounts of native size recombinant spidroins in diverse cell types have been unsuccessful. In these studies, we design and express recombinant miniature black widow MaSp1 spidroins in bacteria that incorporate the N-terminal and C-terminal domain (NTD and CTD), along with varying numbers of codon-optimized internal block repeats. Following spidroin overexpression, we perform quantitative analysis of the bacterial proteome to identify proteins associated with spidroin synthesis. Liquid chromatography with tandem mass spectrometry (LC MS/MS) reveals a list of molecular targets that are differentially expressed after enforced mini-spidroin production. This list included proteins involved in energy management, proteostasis, translation, cell wall biosynthesis, and oxidative stress. Taken together, the purpose of this study was to identify genes within the genome of *Escherichia coli* for molecular targeting to overcome bottlenecks that throttle spidroin overexpression in microorganisms.

## 1. Introduction

Spider silk has extraordinary mechanical properties, including high tensile strength and large extensibility, giving rise to remarkable toughness [1]. Because dragline silk is the easiest to collect, it has been extensively studied by research scientists across the planet. Dragline silk is used as a safety line and for locomotion. Black widow spider (*Latrodectus hesperus*) dragline silk, commonly referred to as major ampullate (MA) silk, has been shown to consist of several distinct proteins. These proteins include major ampullate spidroin 1 (MaSp1), 2 (MaSp2), and 3 (MaSp3), along with low molecular weight cysteine-rich proteins 1, (CRP1), 2 (CRP2), 4 (CRP4), and cysteine-rich secretory protein 3 (CRISP3) [2,3,4,5,6]. The MA spidroins represent large molecular weight proteins (~250 kDa) with internal block repeats flanked by non-repetitive N- and C-terminal domains (NR-NTD and NR-CTDs) [7]. The internal block repeats are composed of highly repetitive short amino acid motifs of GGX, GPGXX, and poly-glycine-alanine (G = glycine, P = proline, and X represents Y = tyrosine, L= leucine, or Q = glutamine) [8], while the NTD and CTD consist of approximately 100 amino acids that are conserved across spidroin family members [9]. This sequence conservation supports the assertion that the NTD and CTD serve critical functional roles during assembly, extrusion, and fiber performance [10]. Despite differences in amino acid sequences, structural biochemical analyses have shown the NTD and CTD both fold into five-helix bundles that facilitate solubility and connectivity of spidroins [11,12,13]. Moreover, the NTD and CTD have been shown to be important in protein dimerization, with implications as chief participants that regulate the spider silk assembly pathway [12,13,14,15]. Specifically, the CTD has been shown to contain a conserved cysteine residue involved in spidroin homo-dimerization [15].

Because of the diversity of spider silk and its outstanding mechanical properties, it has attracted the interests of scientists for utilization in commercial, military, medical, and engineering applications. However, the inability to farm spiders and harvest their natural silk due to their cannibalistic nature, their difficulty in “milking” or removing silk manually, and their venomous nature limit the ability to domesticate spiders for harvesting silk on a large-scale. Thus, given these challenges, scientists have focused on synthetic silk production through wet-spinning or electrospinning methodologies [16,17,18]. Because synthetic fiber production requires vast amounts of recombinant spider silk proteins for spinning, a variety of heterologous protein expression systems have been explored, including goats [19], yeast [20,21], insects [22], plants [23], mammalian cells [24], silkworms and bacteria [25,26,27]; however, none of these systems have produced satisfactory yields of native-size recombinant spidroins on a cost-effective scale to transition into large-scale global manufacturing of synthetic spider silk. In part, the intrinsic molecular features of MA spidroins, which include their long protein chains with internal block repeats, coupled with GC-rich codons that specify large amounts of glycine and alanine, make expression of full-length spidroins in non-spider cells that lack the molecular machinery to transcribe and translate these structural proteins a challenging task. Attempts to devise strategies to genetically alter microbial strains to enhance spidroin expression have been pursued; however, these studies have focused on the expression of the internal block repeats of MA spidroins but have excluded the highly conserved NTD and CTD [28]. Because bacteria represent an economically favorable choice for large-scale native-size recombinant spidroin production, we have focused on the identification of important targets within the bacterial proteome that participate in spidroin biosynthesis, specifically searching for bottleneck proteins that currently throttle high expression of recombinant spidroins. We hypothesize that the discovery of pivotal bacterial “gatekeepers” will provide new avenues to overcome the restriction points of recombinant spidroin expression.

In summary, we have expressed mini-spidroins that include the NTD and CTD, along with various numbers of internal block repeats of MaSp1, and we have performed a global proteomic profiling analysis. The specific goal of our proteomic study was to identify bacterial proteins directly involved in spidroin synthesis, aiming to discover impediments that occur during spidroin overexpression. With the long-term objective of producing large quantities of spidroins, our proteomic profiling following enforced expression of MaSp1 mini-spidroins provides an important list of candidates within the bacterial genome that represent potential targets to advance the development of customized genetically engineered strains of bacteria for optimal spidroin expression.

## 2. Results

### 2.1. MaSp1 NTD-nx-CTD Constructs as Miniature Spidroins (Mini-Spidroins)

**Figure 1:**
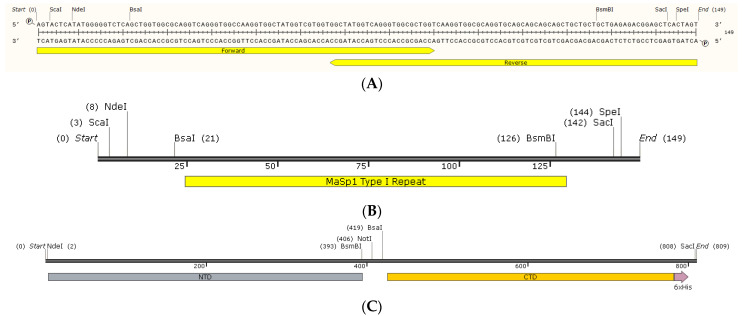
Construction of MaSp1 pET24a-NTD-nx-CTD expression plasmids (n = number of internal block repeats). (**A**) Oligonucleotides used for the creation of the 35 amino acid type I MaSp1 repeat (forward and reverse primers used for annealing and extension), along with engineered restriction sites on the 5′-termini of the oligonucleotides are shown. (**B**) Single copy of the MaSp1 block repeat. (**C**) Fusion of the NTD and CTD coding regions separated by an internal *Bsm*BI and *Bsa*I restriction site for the insertion of MaSp1 block repeats; arrow on CTD represents a 6x-his tag.

One of the major barriers to large-scale synthetic spider silk production is the ability to manufacture vast quantities of recombinant spidroins. Because bacteria divide rapidly and are relatively inexpensive to culture, we investigated the impact of overexpression of mini-spidroins on the bacterial proteome with the goal of identifying important regulators to target for enhanced mini-spidroin recombinant expression. Two different mini-spidroin lengths were created using nucleic acid sequence information from *L. hesperus* MaSp1 (NTD-nx-CTD; n = number of internal block repeats): NTD-2x-CTD and NTD-4x-CTD. A single type I block repeat of MaSp1, the most common ensemble repeat within the primary sequence of MaSp1 [7], was generated by annealing two oligonucleotides that were codon-optimized for bacterial expression, along with the integration of the restriction sites *Bsa*I and *Bsm*BI to facilitate seamless expansion of the internal block repeats (Figure 1A,B). The natural NTD and CTD cDNAs were amplified from a black widow cDNA library prepared from silk-producing glands [29]; these products were also engineered to harbor overlapping sequences, along with the incorporation of internal *Bsm*BI and *Bsa*I sites to allow for insertion of MaSp1 block repeats to create pET24a-NTD-2x-CTD and pET24a-NTD-4x-CTD expression vectors (Figure 1C).

Following the assembly of the MaSp1 NTD-nx-CTD expression vector, the predicted molecular weights of the 2x- and 4x-MaSp1 mini-spidroins were determined to be 31.6 to 37.1 kDa, and their glycine and alanine contents ranged from 15.6 to 21.4% and 21.1 to 22.9%, respectively. The primary amino acid sequences of the NTD-2x-CTD and NTD-4x-CTD mini-spidroins are shown (Table 1).

### 2.2. Expression of MaSp1 Mini-Spidroins in Bacteria

**Figure 2:**
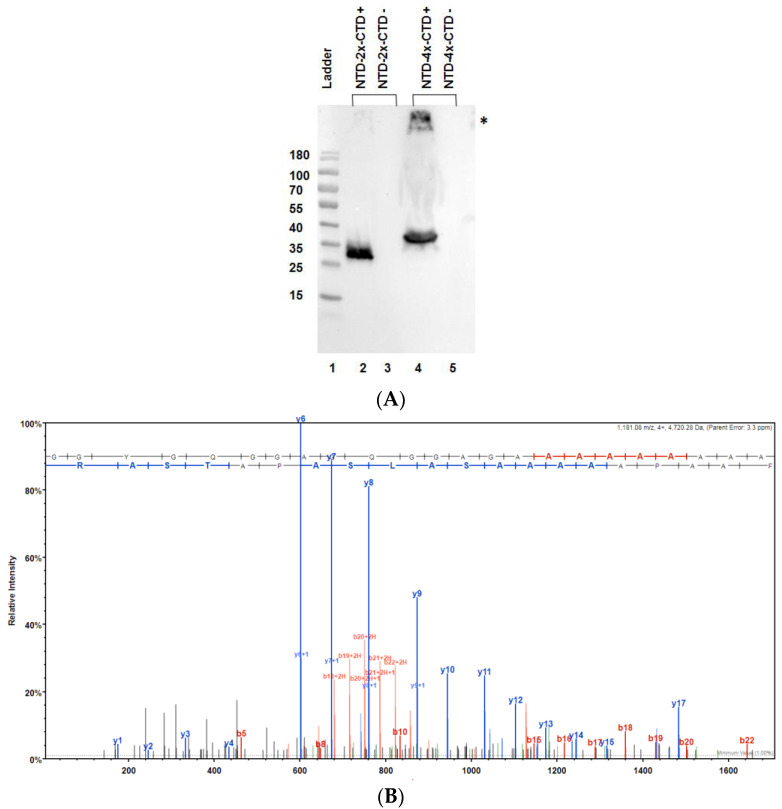
Detection of MaSp1 mini-spidroin expression in bacteria. (**A**) Western blot analysis of crude whole-cell lysates from BL21 DE3 after isopropyl β-D-1-thiogalactopyranoside (IPTG) induction. Lane 1, Thermo Scientific™ PageRuler™ Prestained Protein Ladder in kDa; lane 2, NTD-2x-CTD induced; lane 3, NTD-2x-CTD non-induced; lane 4, NTD-4x-CTD induced; lane 5, NTD-4x-CTD non-induced. Asterisk (*) represents NTD-4x-CTD immunoreactive aggregate complex. (**B**) nLC-MS/MS analysis of BL21(DE3)pLysS whole-cell lysate after expression of mini-spidroins. MS/MS spectrum of precursor ion mass *m*/*z* 4720.28 (M4H+, monoisotopic), whose peptide sequence maps to the MaSp1 block repeat. B- and y-ions represent product ion masses after fragmentation of the precursor ion *m*/*z* 4720.28 using HCD. X- and y-axes represent mass-to-charge values and abundance, respectively.

After assembly of the NTD-nx-CTD MaSp1 gene fusions, the coding regions were verified via DNA sequence analysis, and then the expression vectors were transformed into BL21 (DE3)pLysS cells. Whole cell lysates were collected after recombinant protein induction to confirm mini-spidroin expression via Western blot analysis. Western blot analysis using an anti-histidine mouse monoclonal antibody detected NTD-2x-CTD and NTD-4x-CTD mini-spidroins (Figure 2, lanes 2 and 4). The experimental masses of NTD-2x-CTD and NTD-4x-CTD were consistent with their predicted masses (Table 1). Interestingly, a higher molecular weight immunoreactive species (smear) was also observed in the crude lysate of NTD-4x-CTD expressing cells (Figure 2A, lane 4). This likely represents the inability of the thermal energy, detergent, and reducing agent to dissociate NTD-4x-CTD aggregates, as larger molecular weight spidroins expressed in bacteria have been shown to be difficult to break into monomeric units [28]. To further confirm the presence of MaSp1 mini-spidroin expression in the bacteria, we performed in-solution tryptic digestion followed by nLC-MS/MS analysis using crude bacterial lysates. In both NTD-2x-CTD and NTD-4x-CTD-induced crude bacterial lysates, the MS/MS analysis routinely identified six unique peptides that mapped back to the MaSp1 mini-spidroins. These precursor ions (monoisotopic) corresponded to *m*/*z* values of 764.43 (M2H+), 1149.53 (M2H+), 2764.25 (M3H+), 2831.31 (M4H+), 4702.08 (M4H+), and 4720.28 (M4H+); the precursor ion mass *m*/*z* 4720.28 after fragmentation with HCD is shown (Figure 2B).

Typically, our MS/MS studies yielded approximately 70% sequence coverage of MaSp1 mini-spidroins. We were unable to detect the C-terminus of the mini-spidroins because they lacked an arginine (R) or lysine (K) tryptic cleavage site; protease digestion led to a peptide ion with *m*/*z* of 9510.7 (MH+), which was beyond the quadrupole capabilities of the mass spectrometer (Table 1, underlined region). Taken together, the Western blot analysis and nLC-ESI MS/MS analyses support the expression of both MaSp1 2x and 4x mini-spidroins in the bacteria.

### 2.3. Quantitative Shotgun Proteomic Analysis of Bacteria Expressing MaSp1 Mini-Spidroins

**Figure 3:**
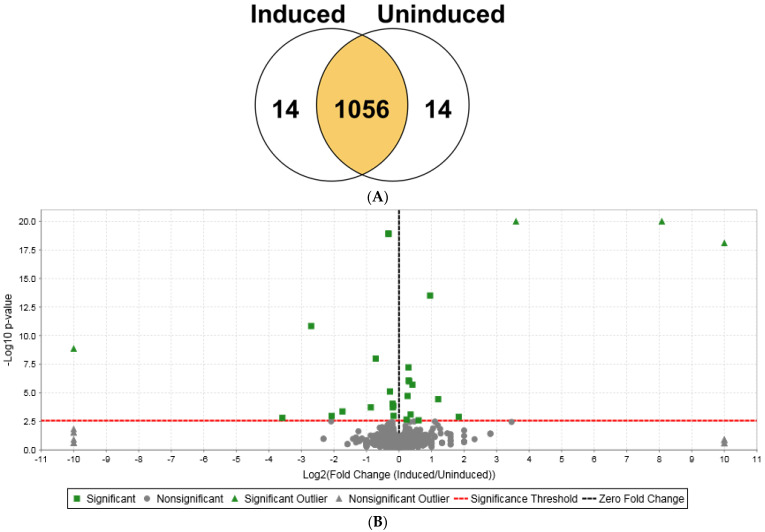
Quantitative proteomic analysis of bacterial proteome after 2 h of NTD-2x-CTD induction versus non-induced control cells. (**A**) Venn diagram of the quantitative profile of proteins identified between induced and non-induced conditions. To assess statistical significance, a Fisher’s exact test with a significance level of *p* = 0.003 and a Benjamini–Hochberg multiple test correction was applied. (**B**) Volcano plot of the mean quantitative profiles of induced versus non-induced proteins expressed by the NTD-2x-CTD constructs. To assess statistical significance, a Fisher’s exact test with a significance level of *p* = 0.003 and a Benjamini–Hochberg multiple test correction was applied. Green squares represent statistically significant proteins, whereas grey circles represent statistically insignificant proteins. Green triangles indicate statistically significant outliers (either very high expression levels or small *p*-values or both), whereas grey triangles depict statistically insignificant outliers. The black dotted vertical line draws the zero-fold change line, whereas the red dotted horizontal line outlines the significance threshold.

To identify bacterial gene products influenced by MaSp1 mini-spidroin expression, we investigated global changes in the bacterial proteome after enforced expression of MaSp1. To reduce the complexities of downstream genetic regulatory networks responding to prolonged IPTG induction, we focused on short-term changes within the bacterial proteome after 2 h of IPTG treatment. Quantitative shotgun proteomic analyses were performed on IPTG-induced and non-induced bacteria carrying NTD-nx-CTD expression vectors. nLC-MS/MS analysis of biological replicates from bacterial cells expressing NTD-2x-CTD mini-spidroins produced 251,735 MS/MS spectra, leading to the identification of 1084 proteins. To compare the relative quantitative profiles of the induced versus non-induced lysates, we analyzed the proteomic data using a Fisher’s exact test (*p*-value less than 0.05) and a Benjamini-Hochberg multiple test correction. Based on the relative quantitative profiles, 28 proteins were differentially expressed, with 14 upregulated and 14 downregulated proteins (Figure 3A,B). All 28 differentially expressed proteins were mathematically statistically significant (Table 2 and Supplementary Table S1); however, we set the boundaries for quantitative significance at ratios of greater than or equal to 1.2 (up and down), similar to other reported studies [30]. During quantitation of the expression data, the Scaffold 5 algorithm defined outliers when fold changes were log_2_ ≥ ±10 (+OLR or −OLR) and/or the *p*-values were 1.00 × 10^−20^ or smaller. In the volcano plot, four proteins were demonstrated to be outliers, including galactoside O-acetyltransferase, beta-galactosidase, MaSp1 NTD-2x-CTD, and D-galactose-binding periplasmic protein (Figure 3A,B; Table 2).

The *p*-values for MaSp1 NTD-2x-CTD and beta-galactosidase were 1.00 × 10^−20^, and their fold-increases were 12.07 and 270.33, respectively (Table 2). Of the 14 induced proteins, 13 were shown to have increases of ≥1.20, while 10 of the downregulated proteins were demonstrated to have decreases of ≥1.20 (Table 2; Supplementary Table S1). Proteomic profiling revealed several proteins involved in lactose or galactose metabolism, including beta-galactosidase, galactoside O-acetyltransferase, D-galactose-binding periplasmic protein, galactose/methyl galactoside import ATP-binding protein, and HTH-type transcriptional regulator GalS, supporting the assertion that the molecular circuitry was responsive to the addition of IPTG, a molecular analog of allolactose. It also demonstrated robust expression of the NTD-2x-CTD spidroin construct (>12 fold), with an increase in the expression of the cellular stress proteins chaperone protein DnaK, chaperone protein HtpG, and catalase-peroxidase (Table 2 and Supplementary Table S1).

**Figure 4:**
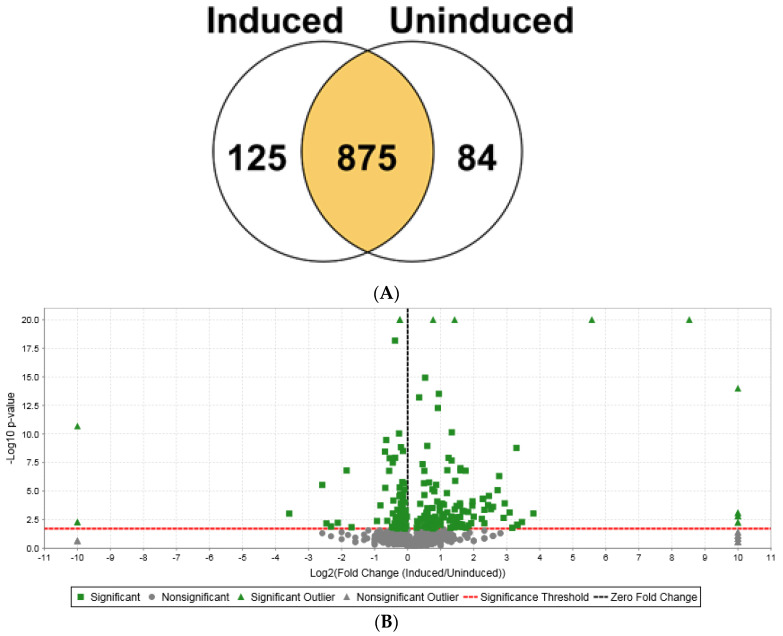
Quantitative shotgun proteomic analysis of bacterial proteome after 2 h of NTD-4x-CTD induction. (**A**) Venn diagram of the quantitative profile of proteins identified between induced and non-induced conditions. To assess statistical significance, a Fisher’s exact test and a Benjamini–Hochberg multiple test correction was applied. (**B**) Volcano plot of the mean quantitative profiles of induced versus non-induced proteins. To assess statistical significance, a Fisher’s exact test with a significance level boundary of *p* = 0.019 and a Benjamini–Hochberg multiple test correction was applied. Green squares represent statistically significant proteins, whereas grey circles represent statistically insignificant proteins. Green triangles depict statistically significant outliers (either very high expression levels or small *p*-values or both), whereas grey triangles indicate statistically insignificant outliers. The black dotted vertical line draws the zero-fold change line, whereas the red dotted horizontal line outlines the significance threshold.

Protein studies employing shotgun proteomics of NTD-4x-CTD-expressing bacterial cell lysates via nLC-ESI MS/MS analysis also led to the identification of 1084 proteins, which corresponded to 259,158 MS/MS spectra. Using similar statistical tools applied to the NTD-2x-CTD construct, we examined the proteomic data by performing a Fisher’s exact test and a Benjamini–Hochberg multiple test correction. A Venn diagram of the relative quantitative values between the induced and non-induced samples showed that 209 proteins were differentially expressed with statistical significance, representing 19.3% of all the identified proteins (Figure 4A,B). Eight hundred and seventy-five proteins showed no change in expression levels (Figure 4A). Of the 209 differentially expressed proteins, 125 were upregulated, while 84 were downregulated (Table 3; Supplementary Table S2). In the volcano plot, there were 9 upregulated and 3 downregulated statistically significant outliers (Figure 4B).

The nine upregulated outliers included galactoside O-acetyltransferase, quinone oxidoreductase 2, chaperedoxin, alanine racemase, uncharacterized protein YiaF, 3-methyl-2-oxobutanoate hydroxymethyltransferase, uncharacterized lipoprotein YajG, MaSp1 NTD-4x-CTD, and beta-galactosidase (Figure 4B, upper right pane; Table 3), while the three downregulated outliers comprised D-galactose-binding periplasmic protein, ribonuclease P protein component, and transcriptional regulator MntR (Figure 4B, upper left pane; Table 3). A total of 50 of the 125 induced proteins displayed ≥3-fold increases, while 75 proteins exhibited fold changes ranging from 1.2 to 2.8 (Table 3 and Supplementary Table S2). Some prominent upregulated proteins included agmatinase (SpeB), cytochrome bo(3) ubiquinol oxidase subunit 1 (CyoA), peroxiredoxin (OsmC), and cold shock protein (CspA). Of the 84 proteins that displayed reduced protein expression, 10 proteins displayed decreases ranging from 3- to 12-fold, while 25 proteins showed reductions in the range of 1.20 to 1.77. Notable downregulated proteins included ATP-dependent RNA helicase (HrpA), glycine-tRNA ligase beta subunit (GlyS), elongation factor 4, aspartate–ammonia ligase, and ribonuclease P protein component—all components of translation (Table 3).

## 3. Discussion

### 3.1. Experimental Strategy

In these studies, we provide new insight into gene expression profile changes experienced by bacteria following the induction of MaSp1 mini-spidroins. To our knowledge, this represents the first study that employs quantitative shotgun proteomics on a global scale to investigate changes in the entire bacterial proteome during spidroin expression, incorporating the NTD, CTD, and internal block repeats—a combination that most closely mimics the natural state of spidroins in arachnids. Enforced expression of MaSp1 mini-spidroins led to the identification of a multitude of differentially expressed proteins within the bacterial proteome. More proteins were identified using the NTD-4x-CTD construct relative to NTD-2x-CTD (209 proteins versus 28, respectively), which likely reflects the propensity of additional internal block repeats to promote aggregation, a current barrier involving the expression of larger molecular weight spidroin constructs in heterologous expression systems. The NTD-4x-CTD protein profiling list included bacterial proteins that were elevated, as well as gene products that were reduced during mini-spidroin expression (Table 3 and Supplementary Table S2). Collectively, our proteomic data provide important insight to delineate bottlenecks that occur during recombinant spidroin expression, offering potential countermeasures to achieve higher expression of spidroins in bacteria.

### 3.2. Identified Targets

In our analysis, as previously mentioned, we observed more statistically significant changes in protein expression for the NTD-4x-CTD construct relative to the NTD-2x-CTD mini-spidroin (Table 2 and Table 3). Examination of the expression of the mini-spidroins via Western blot analysis revealed aggregation of our NTD-4x-CTD construct, a scenario that was not observed when using the NTD-2x-CTD construct (Figure 2, lanes 2 and 4). Evidence to support the responsiveness of the molecular circuitry of the bacteria was demonstrated by IPTG addition, which resulted in the induction of proteins associated with lactose metabolism, including beta-galactosidase, galactoside O-acetyltransferase, D-galactose-binding periplasmic protein, and alpha-galactosidase (Table 2 and Table 3). These expression profile changes were found to be statistically significant. Similar effects of IPTG on bacterial gene expression have been reported via DNA microarray analysis and proteomic studies [31,32].

Deeper inspection of our proteomic data revealed, particularly with the NTD-4x-CTD construct, the appearance of oxidative stress, resource management challenges, and protein aggregation. It has been reported that high-level expression of recombinant proteins in host cells promotes metabolic adaptations, including increased energy production [33]. This, in turn, results in a rise in reactive oxygen species (ROS), including superoxide anion O_2_^−^, hydrogen peroxide, and radical hydroxide HO·, via cellular respiration. In relation to oxidative stress, we observed elevated expression of several proteins involved in energy production, ROS metabolism, and membrane synthesis. These proteomic changes suggest increased respiratory chain activity for energy production by BL21 cells, which is accompanied by the breakdown of additional cellular fuel sources to handle the high energy demands of increased transcription and translation. This assertion is supported by increased levels of quinone oxidoreductase (QorB), a respiratory chain component of *E. coli* that catalyzes the reduction of quinones to increase ATP production. Increased respiration and energy production is also often associated with elevations in ROS. This correlates with increased levels of alkyl hydroperoxide reductase C (AhpC) and peroxiredoxin (OsmC), which are both proteins involved in hydrogen peroxide metabolism [34,35]. We also observed 6.6-fold elevated levels of agmatinase (SpeB), an enzyme that catalyzes the conversion of agmatine to putrescine. Putrescine catabolism is a metabolic response linked to oxidative stress [36]. Both agmatinase and GTP cyclohydrolase 1 type 2 homolog (Ybg1) proteins have also been shown to have functions to protect against oxidative damage [37]. Interestingly, the *speB* gene was identified as one of forty-seven mutated genes in a MaSp1-hosting SoluBL21 strain generated through directed evolution [38]. This mutant strain produced the highest level of spidroins compared to nine other strains. This increase was associated with a single mutation within the *speB* gene, giving rise to a Ser33 to Ala33 alteration in the protein chain [38]. According to this study, the published data did not address whether this mutation resulted in a loss-of-function of SpeB enzymatic activity; therefore, it is difficult to integrate its relationship with our proteomic findings. In our studies, SpeB levels increased during NTD-4x-CTD induction, suggesting that overexpression of our recombinant MaSp1 construct influenced the same stress response pathway. Collectively, both studies seem to pinpoint agmatinase as an important modulator during spidroin expression in bacteria.

Specifically, elevated levels of chaperedoxin (CnoX), a holdase, have been reported to prevent protein aggregation [39]. The elevation of CnoX expression corresponds to the formation of mini-spidroin aggregation, suggesting that CnoX potentially represents an outstanding target to reverse perturbations in proteostasis during spidroin overproduction. During spidroin overexpression, it would seem critical to maintain spidroin solubility (no aggregation or reduced protein mis-folding), which is intricately linked to the viability of bacteria. Additionally, we observed a 3-fold increase in CspA, an RNA-binding protein that is normally induced during a cold-shock response to abrupt shifts to growth conditions at lower temperatures [40]. Interestingly, it has been reported that the growth of bacteria expressing recombinant spidroins is enhanced by shifts to colder temperatures (e.g., 16 °C or 20 °C), yet the underlying mechanism(s) mediating this increase was not elucidated [27,41]. In light of our observations, a shift to lower growth temperatures likely further enhances expression of CspA, assisting in recombinant spidroin synthesis. CspA has been shown to participate in melting of secondary structure within mRNAs, functioning as a thermosensor to facilitate translation [42]. It would appear that elevated levels of CspA after NTD-4x-CTD expression could be assisting in alleviating the secondary structure of MaSp1 mRNA, facilitating increased translation of MaSp1 transcripts. Intriguingly, an analysis of the transcriptome of black widow spiders does predict a protein with a 55% similar identity (64% positive) to bacterial CspA, suggesting a potential linkage and overlapping biological function during spidroin expression in spiders [43]. In the future, we plan on co-overexpressing both CspA and NTD-nx-CTD constructs to improve spidroin production in bacteria. Some of our best candidates identified in our proteomic analysis represent uncharacterized bacterial proteins (accession numbers P0ADK0, P0ADA5, P0AD33, and P77376), which will require further investigation to elucidate their specific roles in spidroin expression (Table 3). Other identified candidates with elevated protein levels are curious, such as alanine racemase, which catalyzes the interconversion of L- and D-alanine [44]. D-alanine is an essential component of the peptidoglycan layer of the bacterial cell wall, which might indicate that overexpression of spidroins in bacteria promotes a form of cell wall stress, influencing cell well assembly. It is also worth noting that we observed a considerable number of additional proteins involved in outer membrane biogenesis, including chain length determinant protein (WzzB), 3-deoxy-manno-octulosonate cytidylyltransferase (KdsB), and phosphoethanolamine transferase (EptA). This supports the hypothesis that overexpression of our mini-spidroins is influencing membrane biogenesis.

If synthetic spider silk is going to be implemented on a global scale, vast quantities of recombinant spidroins will be required for production. These materials must be obtained in a cost-efficient manner, which leads scientists back to improving expression in heterologous recombinant protein systems. Expression in bacteria seems to represent the logical choice. Taken together, our findings reveal several targets for genetic manipulation to establish customized engineered strains of *E. coli*. Moreover, it will be exciting to use these engineered strains to express longer versions of the MaSp1 mini-spidroins that carry more internal block repeats, and our seamless cloning strategy readily allows for expansion to larger constructs (e.g., NTD-8x-CTD, NTD-16x-CTD, NTD-32x-CTD).

## 4. Materials and Methods

### 4.1. Site-Directed Mutagenesis of Parental pET24a Vector

Prior to construction of the MaSp1 expression vectors, site-directed mutagenesis was performed on the parental pET24a cloning vector to destroy the *Bsm*BI restriction site. Three sites were targeted for destruction. These sites were named 923, 2500, and 3627, which reflected their location in base pairs (bp) within the parental pET24a cloning vector (Novagen Inc, Madison, WI, USA). Primers were designed to alter a single nucleotide within the *Bsm*BI restriction sites. The primer sequences utilized for the mutations were as follows: 923F 5′-TGA TTG CGC CTG AGC TAG ACG AAA TAC CGC ATC GC-3′, 923R 5′-GCG ATG CGG TAT TTC GTC TAG CTC AGG CGC AAT CA-3′, 2500F 5′-GAC AAG CTG TGA CCG CCT CCG GGA GCT GCA T-3′, 2500R 5′-ATG CAG CTC CCG GAG GCG GTC ACA GCT TGT C-3′, 3627F 5′-GGT TTT TCT TTT CAC CAG TTA GAC GGG CAA CAG CTG A-3′, and 3627R 5′-TCA GCT GTT GCC CGT CTA ACT GGT GAA AAG AAA AAC C-3′ (underlined nucleotides represent *Bsm*BI mutation). Mutations were introduced using the QuikChange II Site-Directed Mutagenesis kit according to the manufacturer’s instructions (Agilent Technologies, Folsom, CA, USA). Briefly, the pET24a parental vector was denatured, annealed with mutagenic primers, extended with *PfuUltra* DNA polymerase, digested with *Dpn*I, and then transformed into bacterial cells. We first targeted the *Bsm*BI site, positioned at 923 bp. Following mutant strand synthesis and *Dpn*I digestion, the products were transformed into XL1-Blue cells and then plated on media supplemented with 30 μg/mL kanamycin. Colonies were picked and inoculated in Luria broth (LB) and cultured overnight at 37 °C with shaking (200 rpm). To confirm the mutation, purified pET24a plasmid DNA was digested using *Bsm*BI at 55 °C for 2 h, and the products were analyzed using agarose gel electrophoresis. Once the 923 site was mutated, this plasmid was subjugated to a second round of site-directed mutagenesis to alter the second *Bsm*BI site located at 2500 bp. Essentially, all the steps previously described for mutagenesis were identical, but a different primer set was utilized for the mutation of the second *Bsm*BI site within the plasmid. After the *Bsm*BI site at 2500 bp was confirmed to be mutated, the pET24a containing double mutations at 923 and 2500 bp underwent site-directed mutagenesis a final time; a third round to mutate the *Bsm*BI site at position 3627. The final triple-mutated pET24a vector (pET24a triple mutant) was confirmed via restriction digest analysis.

### 4.2. Plasmid Construct Assembly

In order to create the MaSp1 block repeat for multimerization and expression studies, two oligonucleotides were codon-optimized for expression in *Escherichia coli*. Because the desired coding region of the *Latrodectus hesperus* MaSp1 block repeat approached the limitations of primer synthesis, forward and reverse oligonucleotides were designed with short 3′ complementary ends for annealing purposes, while their 5′ single-stranded overhangs allowed for filing by *Taq* DNA polymerase. The MaSp1 forward primer sequence was 5′-**AGT ACT CAT ATG** GG**G GTC TCA GCT G**GT GGC GCA GGT CAG GGT GGC CAA GGT GGC TAT GGT CGT GGT GGC TAT GGT CAG GGT GGC GCT GGT-3′, whereas the MaSp1 reverse primer was 5′-**ACT AGT GAG CTC CGT CTC TCA GC**A GCA GCA GCT GCT GCT GCT GCA CCT GCG CCA CCT TGA CCA GCG CCA CCC TGA CCA TAG CC-3′ (nitrogenous bases that are underlined denote complementary base sequences to facilitate annealing). The forward primer and reverse primers were engineered to incorporate *Sca*I-*Nde*I-*Bsa*I and *Spe*I-*Sac*I-*Bsm*BI sites, respectively (bolded nitrogenous bases). These primers were designed to incorporate unique restriction sites on the 5′-termini of the synthetic MaSp1 block repeat (1×) in order to facilitate subcloning into the mutated pET24a vector. A single block repeat of the MaSp1 coding region was further amplified using two gene-specific primers with the forward and reverse sequences 5′-AGT ACT CAT ATG GGG GTC TCA GCT GG-3′ and 5′-ACT AGT GAG CTC CGT CTC TCA GCA GC-3′, respectively. Following PCR, the products were gel extracted using a QIAquick Gel Extraction Kit according to the manufacturer’s instructions (Qiagen, Hilden, Germany). The gel-extracted DNA fragments were then ligated into pBAD TOPO, and the inserts were subject to DNA sequencing (Sequetech Corporation, Mountain View, CA, USA) to confirm the presence of the synthetic MaSp1 gene. The MaSp1 block repeat was removed by *Nde*I and *Sac*I and ligated into the pET24a triple mutant vector to create pET24a-MaSp1-1x.

### 4.3. Seamless Cloning Strategy

After construction of the pET24a-MaSp1-1x vector, we multimerized the MaSp1 block repeat. In order to eliminate superfluous codon addition at the junctions during the expansion of the MaSp1 synthetic block repeats, we implemented a seamless cloning strategy using three restriction enzymes: *Bsa*I, *Bsm*BI, and *Sac*I; this approach closely followed a previously published study with non-silk genes [45]. Briefly, the pET24a-MaSp1-1x construct was digested with *Bsa*I and *Sac*I, creating a MaSp1-1x block repeat with an intact *Bsm*BI site, while another pET24a-MaSp1-1x construct was digested with *Bsm*BI and *Sac*I, generating compatible ends for ligation and expansion into pET24a-MaSp1-2x. Once the pET24a-MaSp1-2x cloning vector was confirmed, it was used in a similar method as described above to create pET24a-MaSp1-4x. All plasmids containing MaSp1-block expansions were sent for DNA sequencing to confirm that the fusion resulted in seamless joints (Sequetech Corporation, Mountain View, CA, USA).

### 4.4. Construction of NTD and CTD of MaSp1

The cDNAs encoding the NTD and CTD of MaSp1 were amplified with gene-specific primers using a cDNA library prepared from a black widow spider. These primers were designed to incorporate restriction sites on their 5′-termini to facilitate seamless cloning of the codon-optimized MaSp1 block repeats released from pET24a-MaSp1-nx (nx, where n stands for the number of MaSp1 block iterations). The oligonucleotides used to amplify the coding region of the MaSp1 NTD were designed to create restriction sites on the 5′ termini of the forward (*Nde*I; bolded) and reverse primers (*Pvu*II-*Not*I-*Bsm*BI; bolded), respectively. The NTD forward (NTD-F1) and reverse primer (NTD-R1) sequences were 5′-**CAT ATG** CAA GCC AAC ACG CCA TGG T-3′ and 5′-**CAG CTG** AGA CC**G CGG CCG CCG TCT CTC AGC** GGC GTA TAC ATC GTT GGC AGA TGC CTG T-3′, respectively. The forward (CTD-F1) and reverse (CTD-R1) primers used to amplify the MaSp1 CTD were 5′-CCG CTG **AGA GAC GGC GGC CGC GGT CTC** AGC TGC CAG TGG ACC TGG TCA AAT TTA TTA TGG ACC CC-3′ and 5′-**GAG CTC** CTA GTG GTG GTG GTG GTG GTG GGC GAA TGC ATT TTG AAC AGA TTG AG-3′, respectively. The 5′ termini of the CTD forward primer added the restriction sites *Bsm*BI-*Not*I-*Bsa*I, while the reverse primer added a 6x histidine tag and *Sac*I restriction site. Once the MaSp1 cDNAs encoding the NTD and CTD were amplified, they were ligated (separately) into pBAD TOPO to create pBAD-TOPO-NTD and pBAD-TOPO-CTD, respectively. The cDNAs encoding the NTD and CTD were excised from pBAD-TOPO via double restriction digestion using *Nde*I and *Bsm*BI or *Bsm*BI and *Sac*I, respectively, and then they were gel-extracted. Through the incorporation of the *Bsm*BI and *Not*I restriction sites, the 3′ end of the NTD PCR product was complementary to the 5′ end of the CTD PCR product, allowing the NTD segment to be partially annealed to the CTD cDNA, a principle that facilitated the fusion of the two products. Briefly, the gel-extracted digestion products of the NTD- and CTD-encoding segments were combined, denatured for 3 min at 95 °C, then allowed to anneal at 50 °C for 3 min, followed by extension of their overhangs with *Taq* DNA polymerase. After extension, the composite NTD–CTD gene segment was amplified via PCR using the gene-specific NTD-F1 and CTD-R1 primers. The amplified product was ligated into pBAD TOPO and removed via *Nde*I and *Sac*I digestion, followed by ligation into the triple *Bsm*BI mutant pET24a vector, creating pET24a-NTD-CTD.

### 4.5. MaSp1 Mini-Spidroin Design

To assemble the final mini-spidroin expression vectors, the pET24a-NTD-CTD vector (triple mutant) and multiple iterations of pET24a-MaSp1 (e.g., pET24a-MaSp1-2x and 4x) were digested with *Bsa*I and *Bsm*BI. The released MaSp1 block repeat(s) from pET24a-MaSp1-nx were inserted into the pET24a NTD-CTD vector to create pET24a-NTD-MaSp1-nx-CTD; these constructs were often abbreviated as NTD-nx-CTD. All the plasmids were checked via restriction digestion and DNA sequencing analysis.

### 4.6. Recombinant Protein Expression and Western Blot Analysis

For recombinant protein expression in *E. coli*, BL21 (DE3)pLysS chemically competent cells were transformed with pET24a-NTD-MaSp1-nx-CTD plasmids and induced at OD_600_ = 0.5 with 1 mM IPTG. Bacterial cells were grown for 2 h at 37 °C with shaking at 200 rpm. After 2 h, cell densities were determined using visible spectroscopy at 600 nm with a NanoDrop™ 2000c spectrophotometer (Thermo Fisher Scientific, Tracy, CA, USA) and normalized to ensure equivalent bacterial cell densities. Equivalent cell densities were double-pelleted and then prepared for Western blotting and MS/MS analyses. For SDS-PAGE analysis, the double-pellets were re-suspended in protein loading buffer, sonicated, boiled at 95 °C for 10 min, and centrifuged at 16,000× *g* for 10 min. Electrophoresis was performed at 150 V (15 V/cm) for 45 min. After electrophoresis, the proteins were transferred to a PVDF membrane for 7 min at 25 V using the Pierce™ G2 Fast Blotter. The PVDF membrane was blocked for 1 h in 5% powdered milk dissolved in 1× Tris-buffered saline with 0.1% Tween 20^®^ detergent (TBS-T buffer). The antigens were detected using a mouse anti-histidine IgG monoclonal antibody, followed by antigen–antibody detection with a goat anti-mouse IgG secondary antibody conjugated with horse radish peroxidase (HRP). Antigen–antibody complexes were visualized using Thermo Fisher’s chemiluminescent Pierce™ ECL Western blotting substrate.

For MS/MS analyses, two biological replicates of induced and non-induced cells were washed three times with 1× phosphate-buffered saline (PBS pH = 7.5), then subject to in-solution tryptic digestion.

### 4.7. In-Solution Tryptic Digestion

The bacterial cell pellets were lysed with a final concentration of 3M urea (dissolved in 50 mM Tris, pH = 8). After cell lysis, chromosomal DNA was sheared via sonication, followed by reduction of protein disulfide bonds with 5 mM DTT, then sulfhydryl alkylation using 20 mM iodoacetamide (IAA). Excess IAA was quenched via treatment with 20 mM DTT. All incubations with DTT and IAA were performed for 45 min at room temperature. Following reduction and alkylation, the samples were diluted with water to adjust the concentration to 1 M urea. The proteins were digested overnight at 37 °C using 2 μg of trypsin (1:50 ratio of trypsin/substrate). To reduce miscleavage events, the overnight digests were further supplemented with an additional 1 μg of trypsin for 2 h. After digestion, the lysates were centrifuged at 16,000× *g* for 3 min, and the supernatants were transferred to new Eppendorf tubes. Tryptic digestions were terminated with the addition of 1% trifluoroacetic acid (TFA). Prior to desalting and concentration of peptides using Pierce™ C18 Spin columns, the tryptic digests were adjusted to 5% acetonitrile (ACN), then the tryptic peptides were subject to purification according to the manufacturer’s instructions.

### 4.8. Mass Spectrometry and Chromatography

Mass spectrometry analysis was performed using an Orbitrap Fusion™ Tribrid™ mass spectrometer equipped with an Easy-Spray ion source (Thermo Fisher Scientific) operated in data-dependent acquisition mode (DDA) with Xcalibur 4.0 software (Thermo Fisher Scientific). Survey scans were acquired using the Orbitrap (OT) mass detector with a resolution setting of 120,000. Precursor ions were isolated with the quadrupole window set to a range of *m*/*z* 300–1500 Da. The intensity threshold for precursor ion fragmentation was set at 1.0 × 10^5^. Charge states of precursor ions with +2 to +8 were selected for fragmentation. MS/MS data were collected with the OT set to a resolution of 30,000 and fragmentation was performed using HCD with a collision energy of 30%.

For analysis, 250 ng of tryptic digest were injected into the Easy-Spray PepMax^®^ C18 analytical column using a Dionex Ultimate 3000 autosampler. Each biological replicate was injected into the mass spectrometer three times (technical triplicates). The C18 UHPLC column was 75 micron i.d. × 15 cm, 100 Å; Thermo Fisher Scientific, Tracy, CA, USA). A 140 min chromatography run was performed that utilized a customized ACN gradient. Solvents A (water) and B (100% ACN) were both supplemented with 0.1% formic acid. The following 140 min gradient conditions were employed: 2% B for 5 min, 2–22% B for 70 min, 22–38% B for 25 min, 38–95% for 10 min, and 95 to 2% for 30 min. The flow rate was 300 nL per min, and the needle voltage for ionization was set at 1900 Volts.

### 4.9. Data Analysis

The MS/MS spectra were analyzed using Proteome Discoverer 2.4 (Thermo Fisher Scientific, Tracy, CA, USA). A Uniprot protein database for *E. coli* (strain K12; sp_canonical Taxon ID = 83333) was downloaded on 15 April 2022, containing all the SwissProt and Trembl entries. The predicted protein sequences of NTD-nx-CTD of MaSp1 were manually added to the *E. coli* protein database. The following settings were used for the database searches: precursor ion mass tolerance was set to 10 ppm, while the fragment ion mass tolerance was set to 0.02 Da. Variable modifications included carbamidomethylation of cysteine (C), deamination of glutamine and asparagine (Q and N), and oxidation of methionine (M). The Percolator algorithm was used to enhance statistical confidence in protein identifications and was set to a 1% false discovery rate (FDR). After the Proteome Discoverer analysis, data files with msf extensions were imported into Scaffold 5 Q + S (Proteome Software, Seattle, WA, USA) and subject to quantitative analysis. The raw data files can be obtained from the Center for Computational Mass Spectrometry MassIVE (massive.ucsd.edu).

## Figures and Tables

**Table 1:** Amino acid sequences for NTD-nx-CTD (n = 2 and 4) constructs. NTD and CTD are bolded and flank the internal block repeats. Underlined regions denote a tryptic peptide that was unable to be detected using mass spectrometry.

Repeats	Amino Acid Sequence
2×	**MQANTPWSSKANADAFINSFISAASNTGSFSQDQMEDMSLIGNTLMAAMDNMGGRITPSKLQALDMAFASSVAEIAASEGGDLGVTTNAIADALTSAFYQTTGVVNSRFISEIRSLIGMFAQASANDVYAA**GGAGQGGQGGYGRGGYGQGGAGQGGAGAAAAAAAAGGAGQGGQGGYGRGGYGQGGAGQGGAGAAAAAAAAA**SGPGQIYYGPQSVAAPAAAAASALSAPATSARISSHASALLSNGPTNPASISNVISNAVSQISSSNPGASACDVLVQALLELVTALLTIIGSSNIGSVNYDSSGQYAQVVTQSVQNAFAHHHHHH**
4×	**MQANTPWSSKANADAFINSFISAASNTGSFSQDQMEDMSLIGNTLMAAMDNMGGRITPSKLQALDMAFASSVAEIAASEGGDLGVTTNAIADALTSAFYQTTGVVNSRFISEIRSLIGMFAQASANDVYAA**GGAGQGGQGGYGRGGYGQGGAGQGGAGAAAAAAAAGGAGQGGQGGYGRGGYGQGGAGQGGAGAAAAAAAAGGAGQGGQGGYGRGGYGQGGAGQGGAGAAAAAAAAGGAGQGGQGGYGRGGYGQGGAGQGGAGAAAAAAAAA**SGPGQIYYGPQSVAAPAAAAASALSAPATSARISSHASALLSNGPTNPASISNVISNAVSQISSSNPGASACDVLVQALLELVTALLTIIGSSNIGSVNYDSSGQYAQVVTQSVQNAFAHHHHHH**

**Table 2:** List of NTD-2x-CTD statistically significant differentially expressed proteins. Gene names, protein names, and accession numbers (Acc. #) were derived from UniProt. Fold-change ratio (FCR) and outliers (OLR) are also shown. Increased protein levels are represented with a ‘+’ symbol, whereas reduced protein levels are represented by a ‘−’. O.S. = Oxidative stress; asterisks represent components of *lac* operon and lactose metabolism.

Gene	Protein	Acc. #	FCR	*p*-Value	Function
lacA	Galactoside O-acetyltransferase *	P07464	+OLR	7.89 × 10^−19^	Lactose metabolism
lacZ	Beta-galactosidase *	P00722	+OLR +270.33	1.00 × 10^−20^	Lactose metabolism
N/A	MaSp1 NTD-2x-CTD	N/A	+OLR +12.09	1.00 × 10^−20^	Target protein
ybgl	GTP cyclohydrolase 1 type 2 homolog	P0AFP6	+3.57	1.26 × 10^−3^	O.S. resistance
melA	Alpha-galactosidase *	P06720	+2.30	3.59 × 10^−5^	Lactose metabolism
pnp	Polyribonucleotide nucleotidyltransferase	P05055	+1.94	3.08 × 10^−14^	mRNA degradation
slyD	FKBP-type peptidyl-prolyl cis-trans isomerase SlyD	P0A9K9	+1.52	2.52 × 10^−3^	Chaperone
ytfQ	Galactofuranose-binding protein YtfQ	P39325	−1.64	1.04 × 10^−8^	Sugar transport
gatB	PTS system galactitol-specific EIIB component	P37188	−1.82	1.87 × 10^−4^	Sugar transport
pliG	Inhibitor of g-type lysozyme	P76002	−3.33	4.28 × 10^−4^	Inhibitor
mdoG	Glucans biosynthesis protein G	P33136	−4.20	1.06 × 10^−3^	Cell wall synthesis
galS	HTH-type transcriptional regulator GalS	P25748	−6.50	1.47 × 10^−11^	Repressor of *mgl* operon
mglA	Gal./methyl galactoside import ATP-binding protein *	P0AAG8	−12.00	1.53 × 10^−3^	Lactose metabolism
mglB	D-galactose-binding periplasmic protein *	P0AEE5	−OLR	1.39 × 10^−9^	Lactose metabolism

**Table 3:** List of NTD-4x-CTD statistically significant differentially expressed proteins. Gene names, protein names, and accession numbers (Acc. #) were derived from UniProt. Elevated levels are represented with a ‘+’ symbol, whereas diminished levels are represented with a ‘−’ symbol. Asterisks represent components of *lac* operon and lactose metabolism; FCR = fold-change ratio.

Gene	Protein	Acc. #	*p*-Value	FCR	Function
lacA	Galactoside O-acetyltransferase *	P07464	1.03 × 10^−14^	+OLR	Lactose metabolism
qorB	Quinone oxidoreductase 2	P39315	8.38 × 10^−4^	+OLR	Electron transport in respiratory chain
cnoX	Chaperedoxin	P77395	8.38 × 10^−4^	+OLR	Protein deaggregation
dadX	Alanine racemase, catabolic	P29012	5.79 × 10^−3^	+OLR	Isomerizes L- to D-alanine
yiaF	Uncharacterized protein YiaF	P0ADK0	1.60 × 10^−3^	+OLR	Uncharacterized
panB	3-methyl-2-oxobutanoate hydroxymethyltransferase	P31057	1.60 × 10^−3^	+OLR	Pantothenate biosynthesis
yajG	Uncharacterized lipoprotein YajG	P0ADA5	5.79 × 10^−3^	+OLR	Uncharacterized
N/A	MaSp1 NT4XCT	N/A	1.00 × 10^−20^	+OLR	Target protein
lacZ	Beta-galactosidase*	P00722	1.00 × 10^−20^	+OLR	Lactose metabolism
yfcZ	UPF0381 protein YfcZ	P0AD33	9.29 × 10^−4^	+14.00	Uncharacterized
wzzB	Chain length determinant protein	P76372	5.22 × 10^−3^	+11.00	Lipopolysaccharide biosynthesis
kdsB	3-deoxy-manno-octulosonate cytidylyltransferase	P04951	9.18 × 10^−3^	+10.00	Lipopolysaccharide biosynthesis
efeO	Iron uptake system component EfeO	P0AB24	9.18 × 10^−3^	+10.00	Iron uptake
metQ	D-methionine-binding lipoprotein MetQ	P28635	1.68 × 10^−9^	+9.80	Methionine transport system
ydgJ	Uncharacterized oxidoreductase YdgJ	P77376	1.60 × 10^−2^	+9.00	Uncharacterized
osmC	Peroxiredoxin OsmC	P0C0L2	7.66 × 10^−4^	+8.50	Hydroperoxide metabolism
cdd	Cytidine deaminase	P0ABF6	1.18 × 10^−4^	+7.67	Pyrimidine salvage pathway
ycdY	Chaperone protein YcdY	P75915	2.24 × 10^−3^	+7.50	Molybdoenzyme chaperone
cyoB	Cytochrome bo(3) ubiquinol oxidase subunit	P0ABI8	4.95 × 10^−7^	+6.83	Terminal enzyme in respiratory chain
speB	Agmatinase	P60651	8.45 × 10^−6^	+6.60	Putrescine biosynthesis
ushA	Protein UshA	P07024	2.42 × 10^−4^	+6.00	Nucleotidase
yicC	UPF0701 protein YicC	P23839	3.99 × 10^−4^	+5.75	sRNA degradation
ybgl	GTP cyclohydrolase 1 type 2 homolog	P0AFP6	2.71 × 10^−5^	+5.50	Oxidative stress resistance
yfcE	Phosphodiesterase YfcE	P67095	1.68 × 10^−4^	+5.40	Phosphodiesterase
nagA	N-acetylglucosamine-6-phosphate deacetylase	P0AF18	4.38 × 10^−4^	+5.00	Peptidoglycan synthesis
acrB	Multidrug efflux pump subunit AcrB	P31224	6.89 × 10^−3^	+5.00	Transporter
gpr	L-glyceraldehyde 3-phosphate reductase	Q46851	4.80 × 10^−5^	+4.86	Oxidoreductase activity
btsT	Pyruvate/proton symporter BtsT	P39396	4.80 × 10^−5^	+4.86	Pyruvate transport
iscX	Protein IscX	P0C0L9	2.75 × 10^−3^	+4.75	Iron–sulfur cluster assembly
gor	Glutathione reductase	P06715	1.76 × 10^−3^	+4.00	Oxidative stress prevention
fmt	Methionyl-tRNA formyltransferase	P23882	8.04 × 10^−5^	+3.90	Initiation of protein synthesis
gnsA	Protein GnsA	P0AC92	1.92 × 10^−4^	+3.89	Membrane lipid synthesis
maeA	NAD-dependent malic enzyme	P26616	6.69 × 10^−3^	+3.80	Oxidation–reduction
eptA	Phosphoethanolamine transferase EptA	P30845	1.03 × 10^−2^	+3.60	Membrane synthesis
grcA	Autonomous glycyl radical cofactor	P68066	6.75 × 10^−4^	+3.56	Glucose fermentation
zntA	Zinc/cadmium/lead-transporting P-type ATPase	P37617	1.56 × 10^−2^	+3.40	Metal ion transporter
melA	Alpha-galactosidase *	P06720	1.67 × 10^−7^	+3.37	Lactose metabolism
mtlD	Mannitol-1-phosphate 5-dehydrogenase	P09424	5.98 × 10^−4^	+3.27	Oxidoreductase
iscA	Iron-binding protein IscA	P0AAC8	5.55 × 10^−4^	+3.17	Iron-binding protein
rihC	Non-specific ribonucleoside hydrolase RihC	P22564	1.42 × 10^−2^	+3.17	Ribonucleoside cleavage
yhhX	Uncharacterized oxidoreductase YhhX	P46853	8.68 × 10^−3^	+3.14	Uncharacterized
gpml	2,3-bisphosphoglycerate-independent phosphoglycerate mutase	P37689	1.55 × 10^−7^	+3.03	Glycolysis
lpp	Major outer membrane lipoprotein Lpp	P69776	9.98 × 10^−8^	+3.03	Outer membrane lipoprotein
yfcD	Uncharacterized Nudix hydrolase YfcD	P65556	1.28 × 10^−2^	+3.03	Uncharacterized
cspA	Cold shock protein CspA	P0A9X9	1.19 × 10^−4^	+3.00	RNA chaperone
carA	Carbamoyl-phosphate synthase small chain	P0A6F1	1.87 × 10^−4^	+3.00	Arginine and pyrimidine biosynthesis
dcrB	Inner membrane lipoprotein DcrB	P0AEE1	7.85 × 10^−3^	+3.00	Membrane fluidity and homeostasis
hslO	33 kDa chaperonin	P0A6Y5	1.89 × 10^−3^	+3.00	Oxidative stress chaperone
trxC	Thioredoxin 2	P0AGG4	1.28 × 10^−2^	+3.00	Oxidative stress response
malM	Maltose operon periplasmic protein	P03841	1.89 × 10^−3^	+3.00	Maltose transport
spy	Periplasmic chaperone Spy	P77754	1.45 × 10^−2^	−3.25	Chaperone
galS	HTH-type transcriptional regulator GalS	P25748	1.62 × 10^−7^	−3.60	Repression of *mgl* operon
dnaX	DNA polymerase III subunit tau	P06710	6.16 × 10^−3^	−4.33	DNA replication
ppk	Polyphosphate kinase	P0A7B1	1.25 × 10^−2^	−5.00	Polyphosphate synthesis
mglA	Galactose/methyl galactoside import ATP-binding protein MglA *	P0AAG8	6.91 × 10^−3^	−5.50	Galactose import
pliG	Inhibitor of g-type lysozyme	P76002	2.98 × 10^−6^	−6.00	Inhibits g-type lysozyme
hrpA	ATP-dependent RNA helicase HrpA	P43329	9.59 × 10^−4^	−12.00	RNA helicase
mglB	D-galactose-binding periplasmic protein *	P0AEE5	2.11 × 10^−11^	−OLR	Lactose metabolism
rnpA	Ribonuclease P protein component	P0A7Y8	5.44 × 10^−3^	−OLR	tRNA processing
mntR	Transcriptional regulator MntR	P0A9F1	5.44 × 10^−3^	−OLR	Manganese transport regulation

## Data Availability

Data supporting the reported results will be available from the corresponding author.

## Supplementary Materials

The following supporting information can be downloaded at: https://www.mdpi.com/article/10.3390/ijms25063556/s1.
