# Supplementary material for: Quantitative Shotgun Proteomic Analysis of Bacteria after Overexpression of Recombinant Spider Miniature Spidroin, MaSp1
Source: Int J Mol Sci. 2024 Mar 21;25(6):3556. doi: 10.3390/ijms25063556 (PMC10971172; doi:10.3390/ijms25063556)
Supplement: Supplementary file 1 [file ijms-25-03556-s001.zip › Supplementary Table 1.docx]

| **Gene Name** | **Protein Name** | **Accession Number** | **Fold Change Ratios** |
| --- | --- | --- | --- |
| lacA | Galactoside O-acetyltransferase* | P07464 | +OLR |
| lacZ | Beta-galactosidase* | P00722 | +270.33 |
| N/A | MaSp1 NTD-2x-CTD | N/A | +12.09 |
| ybgl | GTP cyclohydrolase 1 type 2 homolog | P0AFP6 | +3.57 |
| melA | Alpha-galactosidase* | P06720 | +2.30 |
| pnp | Polyribonucleotide nucleotidyltransferase | P05055 | +1.94 |
| slyD | FKBP-type peptidyl-prolyl cis-trans isomerase SlyD | P0A9K9 | +1.52 |
| htpG | Chaperone protein HtpG | P0A6Z3 | +1.33 |
| katG | Catalase-peroxidase | P13029 | +1.28 |
| tig | Trigger factor | P0A850 | +1.24 |
| rpsA | 30S ribosomal protein S1 | P0AG67 | +1.23 |
| dnaK | Chaperone protein DnaK | P0A6Y8 | +1.22 |
| rplL | 50S ribosomal protein L7/L12 | P0A7K2 | +1.20 |
| ahpC | Alkyl hydroperoxide reductase C | P0AE08 | +1.17 |
| rpoC | DNA-directed RNA polymerase subunit beta | P0A8T7 | -1.12 |
| tnaA | Tryptophanase | P0A853 | -1.13 |
| gapA | Glyceraldehyde-3-phosphate dehydrogenase A | P0A9B2 | -1.14 |
| hupB | DNA-binding protein HU-beta | P0ACF4 | -1.15 |
| lpdA | Dihydrolipoyl dehydrogenase | P0A9P0 | -1.21 |
| tufB | Elongation factor Tu 2 | P0CE48 | -1.25 |
| tufA | Elongation factor Tu 1 | P0CE47 | -1.25 |
| ytfQ | Galactofuranose-binding protein YtfQ | P39325 | -1.64 |
| gatB | PTS system galactitol-specific EIIB component | P37188 | -1.82 |
| pliG | Inhibitor of g-type lysozyme | P76002 | -3.33 |
| mdoG | Glucans biosynthesis protein G | P33136 | -4.20 |
| galS | HTH-type transcriptional regulator GalS | P25748 | -6.50 |
| mglA | Galactose/methyl galactoside import ATP-binding protein MglA* | P0AAG8 | -12.00 |
| mglB | D-galactose-binding periplasmic protein* | P0AEE5 | -OLR |
