# Supplementary material for: Quantitative Shotgun Proteomic Analysis of Bacteria after Overexpression of Recombinant Spider Miniature Spidroin, MaSp1
Source: Int J Mol Sci. 2024 Mar 21;25(6):3556. doi: 10.3390/ijms25063556 (PMC10971172; doi:10.3390/ijms25063556)
Supplement: Supplementary file 1 [file ijms-25-03556-s001.zip › Supplementary Table 1.pdf]

Gene Name	Protein Name	Accession Number	Fold Change Ratios
lacA	Galactoside O-acetyltransferase*	P07464	+OLR
lacZ	Beta-galactosidase*	P00722	+270.33
N/A	MaSp1 NTD-2x-CTD	N/A	+12.09
ybgl	GTP cyclohydrolase 1 type 2 homolog	P0AFP6	+3.57
melA	Alpha-galactosidase*	P06720	+2.30
pnp	Polyribonucleotide nucleotidyltransferase	P05055	+1.94
slyD	FKBP-type peptidyl-prolyl cis-trans isomerase SlyD	P0A9K9	+1.52
htpG	Chaperone protein HtpG	P0A6Z3	+1.33
katG	Catalase-peroxidase	P13029	+1.28
tig	Trigger factor	P0A850	+1.24
rpsA	30S ribosomal protein S1	P0AG67	+1.23
dnaK	Chaperone protein DnaK	P0A6Y8	+1.22
rplL	50S ribosomal protein L7/L12	P0A7K2	+1.20
ahpC	Alkyl hydroperoxide reductase C	P0AE08	+1.17
rpoC	DNA-directed RNA polymerase subunit beta	P0A8T7	-1.12
tnaA	Tryptophanase	P0A853	-1.13
gapA	Glyceraldehyde-3-phosphate dehydrogenase A	P0A9B2	-1.14
hupB	DNA-binding protein HU-beta	P0ACF4	-1.15
lpdA	Dihydrolipoyl dehydrogenase	P0A9P0	-1.21
tufB	Elongation factor Tu 2	P0CE48	-1.25
tufA	Elongation factor Tu 1	P0CE47	-1.25
ytfQ	Galactofuranose-binding protein YtfQ	P39325	-1.64
gatB	PTS system galactitol-specific EIIB component	P37188	-1.82
pliG	Inhibitor of g-type lysozyme	P76002	-3.33
mdoG	Glucans biosynthesis protein G	P33136	-4.20
galS	HTH-type transcriptional regulator GalS	P25748	-6.50
mglA	Galactose/methyl galactoside import ATP-binding protein MglA*	P0AAG8	-12.00
mglB	D-galactose-binding periplasmic protein*	P0AEE5	-OLR
