# Supplementary material for: Quantitative Shotgun Proteomic Analysis of Bacteria after Overexpression of Recombinant Spider Miniature Spidroin, MaSp1
Source: Int J Mol Sci. 2024 Mar 21;25(6):3556. doi: 10.3390/ijms25063556 (PMC10971172; doi:10.3390/ijms25063556)
Supplement: Supplementary file 1 [file ijms-25-03556-s001.zip › Supplementary Table 2.docx]

| **Gene Name** | **Protein Name** | **Accession Number** | **Fold Change Ratios** |
| --- | --- | --- | --- |
| lacA | Galactoside O-acetyltransferase* | P07464 | +OLR |
| qorB | Quinone oxidoreductase 2 | P39315 | +OLR |
| cnoX | Chaperedoxin | P77395 | +OLR |
| dadX | Alanine racemase, catabolic | P29012 | +OLR |
| yiaF | Uncharacterized protein YiaF | P0ADK0 | +OLR |
| panB | 3-methyl-2-oxobutanoate hydroxymethyltransferase | P31057 | +OLR |
| yajG | Uncharacterized lipoprotein YajG | P0ADA5 | +OLR |
| N/A | MaSp1 NTD-4x-CTD | N/A | +OLR |
| lacZ | Beta-galactosidase* | P00722 | +OLR |
| yfcZ | UPF0381 protein YfcZ | P0AD33 | +14.00 |
| wzzB | Chain length determinant protein | P76372 | +11.00 |
| kdsB | 3-deoxy-manno-octulosonate cytidylyltransferase | P04951 | +10.00 |
| efeO | Iron uptake system component EfeO | P0AB24 | +10.00 |
| metQ | D-methionine-binding lipoprotein MetQ | P28635 | +9.80 |
| ydgJ | Uncharacterized oxidoreductase YdgJ | P77376 | +9.00 |
| osmC | Peroxiredoxin OsmC | P0C0L2 | +8.50 |
| cdd | Cytidine deaminase | P0ABF6 | +7.67 |
| ycdY | Chaperone protein YcdY | P75915 | +7.50 |
| cyoB | Cytochrome bo(3) ubiquinol oxidase subunit 1 | P0ABI8 | +6.83 |
| speB | Agmatinase | P60651 | +6.60 |
| ushA | Protein UshA | P07024 | +6.00 |
| yicC | UPF0701 protein YicC | P23839 | +5.75 |
| ybgl | GTP cyclohydrolase 1 type 2 homolog | P0AFP6 | +5.50 |
| yfcE | Phosphodiesterase YfcE | P67095 | +5.40 |
| nagA | N-acetylglucosamine-6-phosphate deacetylase | P0AF18 | +5.00 |
| acrB | Multidrug efflux pump subunit AcrB | P31224 | +5.00 |
| gpr | L-glyceraldehyde 3-phosphate reductase | Q46851 | +4.86 |
| btsT | Pyruvate/proton symporter BtsT | P39396 | +4.86 |
| iscX | Protein IscX | P0C0L9 | +4.75 |
| gor | Glutathione reductase | P06715 | +4.00 |
| fmt | Methionyl-tRNA formyltransferase | P23882 | +3.90 |
| gnsA | Protein GnsA | P0AC92 | +3.89 |
| maeA | NAD-dependent malic enzyme | P26616 | +3.80 |
| eptA | Phosphoethanolamine transferase EptA | P30845 | +3.60 |
| grcA | Autonomous glycyl radical cofactor | P68066 | +3.56 |
| zntA | Zinc/cadmium/lead-transporting P-type ATPase | P37617 | +3.40 |
| melA | Alpha-galactosidase* | P06720 | +3.37 |
| mtlD | Mannitol-1-phosphate 5-dehydrogenase | P09424 | +3.27 |
| iscA | Iron-binding protein IscA | P0AAC8 | +3.17 |
| rihC | Non-specific ribonucleoside hydrolase RihC | P22564 | +3.17 |
| yhhX | Uncharacterized oxidoreductase YhhX | P46853 | +3.14 |
| gpml | 2,3-bisphosphoglycerate-independent phosphoglycerate mutase | P37689 | +3.03 |
| lpp | Major outer membrane lipoprotein Lpp | P69776 | +3.03 |
| yfcD | Uncharacterized Nudix hydrolase YfcD | P65556 | +3.03 |
| cspA | Cold shock protein CspA | P0A9X9 | +3.00 |
| carA | Carbamoyl-phosphate synthase small chain | P0A6F1 | +3.00 |
| dcrB | Inner membrane lipoprotein DcrB | P0AEE1 | +3.00 |
| hslO | 33 kDa chaperonin | P0A6Y5 | +3.00 |
| trxC | Thioredoxin 2 | P0AGG4 | +3.00 |
| malM | Maltose operon periplasmic protein | P03841 | +3.00 |
| cyoA | Cytochrome bo(3) ubiquinol oxidase subunit 2 | P0ABJ1 | +2.82 |
| frdB | Fumarate reductase iron-sulfur subunit | P0AC47 | +2.79 |
| gloA | Lactoylglutathione lyase | P0AC81 | +2.77 |
| fldA | Flavodoxin 1 | P61949 | +2.71 |
| curA | NADPH-dependent curcumin reductase | P76113 | +2.71 |
| gatC | PTS system galactitol-specific EIIC component | P69831 | +2.70 |
| pepQ | Xaa-Pro dipeptidase | P21165 | +2.68 |
| pnp | Polyribonucleotide nucleotidyltransferase | P05055 | +2.68 |
| guaA | GMP synthase [glutamine-hydrolyzing] | P04079 | +2.67 |
| yjjA | Uncharacterized protein YjjA | P18390 | +2.67 |
| pgi | Glucose-6-phosphate isomerase | P0A6T1 | +2.53 |
| ppiD | Periplasmic chaperone PpiD | P0ADY1 | +2.52 |
| fruB | Multiphosphoryl transfer protein | P69811 | +2.50 |
| elbB | Glyoxalase ElbB | P0ABU5 | +2.50 |
| ycaR | UPF0434 protein YcaR | P0AAZ7 | +2.45 |
| arcB | Aerobic respiration control sensor protein ArcB | P0AEC3 | +2.41 |
| glnS | Glutamine--tRNA ligase | P00962 | +2.36 |
| eda | KHG/KDPG aldolase | P0A955 | +2.30 |
| ybiB | Uncharacterized protein YbiB | P30177 | +2.30 |
| iscU | Iron-sulfur cluster assembly scaffold protein IscU | P0ACD4 | +2.28 |
| hldD | ADP-L-glycero-D-manno-heptose-6-epimerase | P67910 | +2.14 |
| osmY | Osmotically-inducible protein Y | P0AFH8 | +2.14 |
| secD | Protein translocase subunit SecD | P0AG90 | +2.06 |
| arnB | UDP-4-amino-4-deoxy-L-arabinose--oxoglutarate aminotransferase | P77690 | +2.05 |
| ygiC | Putative acid--amine ligase YgiC | P0ADT5 | +2.00 |
| gpsA | Glycerol-3-phosphate dehydrogenase [NAD(P)+] | P0A6S7 | +2.00 |
| yjdM | Protein YjdM | P0AFJ1 | +1.96 |
| elaB | Protein ElaB | P0AEH5 | +1.95 |
| katG | Catalase-peroxidase | P13029 | +1.93 |
| acpP | Acyl carrier protein | P0A6A8 | +1.89 |
| pal | Peptidoglycan-associated lipoprotein | P0A912 | +1.86 |
| deoD | Purine nucleoside phosphorylase DeoD-type | P0ABP8 | +1.82 |
| cmk | Cytidylate kinase | P0A6I0 | +1.80 |
| eptC | Phosphoethanolamine transferase EptC | P0CB39 | +1.79 |
| yibT | Uncharacterized protein YibT | Q2M7R5 | +1.79 |
| glpQ | Glycerophosphodiester phosphodiesterase, periplasmic | P09394 | +1.76 |
| pdxJ | Pyridoxine 5'-phosphate synthase | P0A794 | +1.76 |
| maeB | NADP-dependent malic enzyme | P76558 | +1.75 |
| rplL | 50S ribosomal protein L7/L12 | P0A7K2 | +1.71 |
| glpD | Aerobic glycerol-3-phosphate dehydrogenase | P13035 | +1.70 |
| dapA | 4-hydroxy-tetrahydrodipicolinate synthase | P0A6L2 | +1.69 |
| prlC | Oligopeptidase A | P27298 | +1.68 |
| cpdB | 2',3'-cyclic-nucleotide 2'-phosphodiesterase/3'-nucleotidase | P08331 | +1.68 |
| ribE | 6,7-dimethyl-8-ribityllumazine synthase | P61714 | +1.65 |
| atpF | ATP synthase subunit b | P0ABA0 | +1.63 |
| rpe | Ribulose-phosphate 3-epimerase | P0AG07 | +1.61 |
| hdhA | 7alpha-hydroxysteroid dehydrogenase | P0AET8 | +1.60 |
| ridA | 2-iminobutanoate/2-iminopropanoate deaminase | P0AF93 | +1.59 |
| gmhA | Phosphoheptose isomerase | P63224 | +1.59 |
| aspC | Aspartate aminotransferase | P00509 | +1.57 |
| trxA | Thioredoxin 1 | P0AA25 | +1.56 |
| yjbJ | UPF0337 protein YjbJ | P68206 | +1.53 |
| acrA | Multidrug efflux pump subunit AcrA | P0AE06 | +1.52 |
| zapB | Cell division protein ZapB | P0AF36 | +1.52 |
| tpx | Thiol peroxidase | P0A862 | +1.51 |
| pgk | Phosphoglycerate kinase | P0A799 | +1.51 |
| ptsl | Phosphoenolpyruvate-protein phosphotransferase | P08839 | +1.49 |
| nusA | Transcription termination/antitermination protein NusA | P0AFF6 | +1.49 |
| pspA | Phage shock protein A | P0AFM6 | +1.48 |
| cydA | Cytochrome bd-I ubiquinol oxidase subunit 1 | P0ABJ9 | +1.47 |
| gnd | 6-phosphogluconate dehydrogenase, decarboxylating | P00350 | +1.46 |
| dnaK | Chaperone protein DnaK | P0A6Y8 | +1.44 |
| sdhB | Succinate dehydrogenase iron-sulfur subunit | P07014 | +1.42 |
| htpG | Chaperone protein HtpG | P0A6Z3 | +1.42 |
| ackA | Acetate kinase | P0A6A3 | +1.42 |
| ahpC | Alkyl hydroperoxide reductase C | P0AE08 | +1.42 |
| tsf | Elongation factor Ts | P0A6P1 | +1.41 |
| rpsA | 30S ribosomal protein S1 | P0AG67 | +1.37 |
| oppA | Periplasmic oligopeptide-binding protein | P23843 | +1.34 |
| ompA | Outer membrane protein A | P0A910 | +1.31 |
| aceF | Dihydrolipoyllysine-residue acetyltransferase component of pyruvate dehydrogenase complex | P06959 | +1.30 |
| groEL | Chaperonin GroEL | P0A6F5 | +1.27 |
| putA | Bifunctional protein PutA | P09546 | +1.25 |
| eno | Enolase | P0A6P9 | +1.22 |
| rplJ | 50S ribosomal protein L10 | P0A7J3 | +1.21 |
| rpsC | 30S ribosomal protein S3 | P0A7V3 | -1.02 |
| sucD | Succinate--CoA ligase [ADP-forming] subunit alpha | P0AGE9 | -1.03 |
| rplI | 50S ribosomal protein L9 | P0A7R1 | -1.04 |
| rplE | 50S ribosomal protein L5 | P62399 | -1.05 |
| gatD | Galactitol 1-phosphate 5-dehydrogenase | P0A9S3 | -1.05 |
| rplR | 50S ribosomal protein L18 | P0C018 | -1.06 |
| rho | Transcription termination factor Rho | P0AG30 | -1.06 |
| gapA | Glyceraldehyde-3-phosphate dehydrogenase A | P0A9B2 | -1.06 |
| raiA | Ribosome-associated inhibitor A | P0AD49 | -1.06 |
| rplM | 50S ribosomal protein L13 | P0AA10 | -1.06 |
| ptsH | Phosphocarrier protein HPr | P0AA04 | -1.06 |
| rpsL | 30S ribosomal protein S12 | P0A7S3 | -1.07 |
| rpsS | 30S ribosomal protein S19 | P0A7U3 | -1.07 |
| infC | Translation initiation factor IF-3 | P0A707 | -1.07 |
| pta | Phosphate acetyltransferase | P0A9M8 | -1.07 |
| tnaA | Tryptophanase | P0A853 | -1.07 |
| pyrG | CTP synthase | P0A7E5 | -1.08 |
| rpmG | 50S ribosomal protein L33 | P0A7N9 | -1.08 |
| rplB | 50S ribosomal protein L2 | P60422 | -1.08 |
| rpmC | 50S ribosomal protein L29 | P0A7M6 | -1.08 |
| aceA | Isocitrate lyase | P0A9G6 | -1.09 |
| rpsE | 30S ribosomal protein S5 | P0A7W1 | -1.10 |
| ydcH | Uncharacterized protein YdcH | P0ACW6 | -1.10 |
| hns | DNA-binding protein H-NS | P0ACF8 | -1.10 |
| rpsR | 30S ribosomal protein S18 | P0A7T7 | -1.10 |
| rplX | 50S ribosomal protein L24 | P60624 | -1.11 |
| rplV | 50S ribosomal protein L22 | P61175 | -1.11 |
| rpsF | 30S ribosomal protein S6 | P02358 | -1.11 |
| hslU | ATP-dependent protease ATPase subunit HslU | P0A6H5 | -1.11 |
| rplF | 50S ribosomal protein L6 | P0AG55 | -1.12 |
| skp | Chaperone protein Skp | P0AEU7 | -1.13 |
| glyA | Serine hydroxymethyltransferase | P0A825 | -1.13 |
| rpsG | 30S ribosomal protein S7 | P02359 | -1.13 |
| dapD | 2,3,4,5-tetrahydropyridine-2,6-dicarboxylate N-succinyltransferase | P0A9D8 | -1.13 |
| degP | Periplasmic serine endoprotease DegP | P0C0V0 | -1.14 |
| rplY | 50S ribosomal protein L25 | P68919 | -1.14 |
| rpsJ | 30S ribosomal protein S10 | P0A7R5 | -1.14 |
| rpsD | 30S ribosomal protein S4 | P0A7V8 | -1.15 |
| rpsT | 30S ribosomal protein S20 | P0A7U7 | -1.15 |
| rpsI | 30S ribosomal protein S9 | P0A7X3 | -1.16 |
| rpsH | 30S ribosomal protein S8 | P0A7W7 | -1.17 |
| rplW | 50S ribosomal protein L23 | P0ADZ0 | -1.17 |
| stpA | DNA-binding protein StpA | P0ACG1 | -1.18 |
| tufB | Elongation factor Tu 2 | P0CE48 | -1.18 |
| tufA | Elongation factor Tu 1 | P0CE47 | -1.18 |
| pykA | Pyruvate kinase II | P21599 | -1.18 |
| rplP | 50S ribosomal protein L16 | P0ADY7 | -1.18 |
| rpmB | 50S ribosomal protein L28 | P0A7M2 | -1.19 |
| cysK | Cysteine synthase A | P0ABK5 | -1.19 |
| rpoC | DNA-directed RNA polymerase subunit beta | P0A8T7 | -1.20 |
| glyS | Glycine--tRNA ligase beta subunit | P00961 | -1.20 |
| rpmI | 50S ribosomal protein L35 | P0A7Q1 | -1.21 |
| rpmH | 50S ribosomal protein L34 | P0A7P5 | -1.24 |
| eco | Ecotin | P23827 | -1.24 |
| glnH | Glutamine-binding periplasmic protein | P0AEQ3 | -1.27 |
| accC | Biotin carboxylase | P24182 | -1.28 |
| csrA | Carbon storage regulator | P69913 | -1.29 |
| clpA | ATP-dependent Clp protease ATP-binding subunit ClpA | P0ABH9 | -1.30 |
| rplS | 50S ribosomal protein L19 | P0A7K6 | -1.30 |
| hupA | DNA-binding protein HU-alpha | P0ACF0 | -1.30 |
| mreB | Cell shape-determining protein MreB | P0A9X4 | -1.32 |
| argG | Argininosuccinate synthase | P0A6E4 | -1.35 |
| ydgH | Protein YdgH | P76177 | -1.35 |
| ppiA | Peptidyl-prolyl cis-trans isomerase A | P0AFL3 | -1.37 |
| rplN | 50S ribosomal protein L14 | P0ADY3 | -1.37 |
| srmB | ATP-dependent RNA helicase SrmB | P21507 | -1.39 |
| crp | cAMP-activated global transcriptional regulator CRP | P0ACJ8 | -1.46 |
| rpmF | 50S ribosomal protein L32 | P0A7N4 | -1.48 |
| cra | Catabolite repressor/activator | P0ACP1 | -1.53 |
| ytfQ | Galactofuranose-binding protein YtfQ | P39325 | -1.57 |
| rnr | Ribonuclease R | P21499 | -1.60 |
| rpsM | 30S ribosomal protein S13 | P0A7S9 | -1.61 |
| asnA | Aspartate--ammonia ligase | P00963 | -1.77 |
| lepA | Elongation factor 4 | P60785 | -1.90 |
| spy | Periplasmic chaperone Spy | P77754 | -3.25 |
| galS | HTH-type transcriptional regulator GalS | P25748 | -3.60 |
| dnaX | DNA polymerase III subunit tau | P06710 | -4.33 |
| ppk | Polyphosphate kinase | P0A7B1 | -5.00 |
| mglA | Galactose/methyl galactoside import ATP-binding protein MglA* | P0AAG8 | -5.50 |
| pliG | Inhibitor of g-type lysozyme | P76002 | -6.00 |
| hrpA | ATP-dependent RNA helicase HrpA | P43329 | -12.00 |
| mglB | D-galactose-binding periplasmic protein* | P0AEE5 | -OLR |
| rnpA | Ribonuclease P protein component | P0A7Y8 | -OLR |
| mntR | Transcriptional regulator MntR | P0A9F1 | -OLR |
