# Supplementary material for: Quantitative Shotgun Proteomic Analysis of Bacteria after Overexpression of Recombinant Spider Miniature Spidroin, MaSp1
Source: Int J Mol Sci. 2024 Mar 21;25(6):3556. doi: 10.3390/ijms25063556 (PMC10971172; doi:10.3390/ijms25063556)
Supplement: Supplementary file 1 [file ijms-25-03556-s001.zip › Supplementary Table 2.pdf]

Gene Name	Protein Name	Accession Number	Fold Change Ratios
lacA	Galactoside O-acetyltransferase*	P07464	+OLR
qorB	Quinone oxidoreductase 2	P39315	+OLR
cnoX	Chaperedoxin	P77395	+OLR
dadX	Alanine racemase, catabolic	P29012	+OLR
yiaF	Uncharacterized protein YiaF	P0ADK0	+OLR
panB	3-methyl-2-oxobutanoate hydroxymethyltransferase	P31057	+OLR
yajG	Uncharacterized lipoprotein YajG	P0ADA5	+OLR
N/A	MaSp1 NTD-4x-CTD	N/A	+OLR
lacZ	Beta-galactosidase*	P00722	+OLR
yfcZ	UPF0381 protein YfcZ	P0AD33	+14.00
wzzB	Chain length determinant protein	P76372	+11.00
kdsB	3-deoxy-manno-octulosonate cytidylyltransferase	P04951	+10.00
efeO	Iron uptake system component EfeO	P0AB24	+10.00
metQ	D-methionine-binding lipoprotein MetQ	P28635	+9.80
ydgJ	Uncharacterized oxidoreductase YdgJ	P77376	+9.00
osmC	Peroxiredoxin OsmC	P0C0L2	+8.50
cdd	Cytidine deaminase	P0ABF6	+7.67
ycdY	Chaperone protein YcdY	P75915	+7.50
cyoB	Cytochrome bo(3) ubiquinol oxidase subunit 1	P0ABI8	+6.83
speB	Agmatinase	P60651	+6.60
ushA	Protein UshA	P07024	+6.00
yicC	UPF0701 protein YicC	P23839	+5.75
ybgl	GTP cyclohydrolase 1 type 2 homolog	P0AFP6	+5.50
yfcE	Phosphodiesterase YfcE	P67095	+5.40
nagA	N-acetylglucosamine-6-phosphate deacetylase	P0AF18	+5.00
acrB	Multidrug efflux pump subunit AcrB	P31224	+5.00
gpr	L-glyceraldehyde 3-phosphate reductase	Q46851	+4.86
btsT	Pyruvate/proton symporter BtsT	P39396	+4.86
iscX	Protein IscX	P0C0L9	+4.75
gor	Glutathione reductase	P06715	+4.00
fmt	Methionyl-tRNA formyltransferase	P23882	+3.90
gnsA	Protein GnsA	P0AC92	+3.89
maeA	NAD-dependent malic enzyme	P26616	+3.80
eptA	Phosphoethanolamine transferase EptA	P30845	+3.60
grcA	Autonomous glycyl radical cofactor	P68066	+3.56
zntA	Zinc/cadmium/lead-transporting P-type ATPase	P37617	+3.40
melA	Alpha-galactosidase*	P06720	+3.37
mtlD	Mannitol-1-phosphate 5-dehydrogenase	P09424	+3.27
iscA	Iron-binding protein IscA	P0AAC8	+3.17
rihC	Non-specific ribonucleoside hydrolase RihC	P22564	+3.17
yhhX	Uncharacterized oxidoreductase YhhX	P46853	+3.14

gpml	2,3-bisphosphoglycerate-independent phosphoglycerate mutase	P37689	+3.03
lpp	Major outer membrane lipoprotein Lpp	P69776	+3.03
yfcD	Uncharacterized Nudix hydrolase YfcD	P65556	+3.03
cspA	Cold shock protein CspA	P0A9X9	+3.00
carA	Carbamoyl-phosphate synthase small chain	P0A6F1	+3.00
dcrB	Inner membrane lipoprotein DcrB	P0AEE1	+3.00
hslO	33 kDa chaperonin	P0A6Y5	+3.00
trxC	Thioredoxin 2	P0AGG4	+3.00
malM	Maltose operon periplasmic protein	P03841	+3.00
cyoA	Cytochrome bo(3) ubiquinol oxidase subunit 2	P0ABJ1	+2.82
frdB	Fumarate reductase iron-sulfur subunit	P0AC47	+2.79
gloA	Lactoylglutathione lyase	P0AC81	+2.77
fldA	Flavodoxin 1	P61949	+2.71
curA	NADPH-dependent curcumin reductase	P76113	+2.71
gatC	PTS system galactitol-specific EIIC component	P69831	+2.70
pepQ	Xaa-Pro dipeptidase	P21165	+2.68
pnp	Polyribonucleotide nucleotidyltransferase	P05055	+2.68
guaA	GMP synthase [glutamine-hydrolyzing]	P04079	+2.67
yjjA	Uncharacterized protein YjjA	P18390	+2.67
pgi	Glucose-6-phosphate isomerase	P0A6T1	+2.53
ppiD	Periplasmic chaperone PpiD	P0ADY1	+2.52
fruB	Multiphosphoryl transfer protein	P69811	+2.50
elbB	Glyoxalase ElbB	P0ABU5	+2.50
ycaR	UPF0434 protein YcaR	P0AAZ7	+2.45
arcB	Aerobic respiration control sensor protein ArcB	P0AEC3	+2.41
glnS	Glutamine--tRNA ligase	P00962	+2.36
eda	KHG/KDPG aldolase	P0A955	+2.30
ybiB	Uncharacterized protein YbiB	P30177	+2.30
iscU	Iron-sulfur cluster assembly scaffold protein IscU	P0ACD4	+2.28
hldD	ADP-L-glycero-D-manno-heptose-6-epimerase	P67910	+2.14
osmY	Osmotically-inducible protein Y	P0AFH8	+2.14
secD	Protein translocase subunit SecD	P0AG90	+2.06
arnB	UDP-4-amino-4-deoxy-L-arabinose--oxoglutarate aminotransferase	P77690	+2.05
ygiC	Putative acid--amine ligase YgiC	P0ADT5	+2.00
gpsA	Glycerol-3-phosphate dehydrogenase [NAD(P)+]	P0A6S7	+2.00
yjdM	Protein YjdM	P0AFJ1	+1.96
elaB	Protein ElaB	P0AEH5	+1.95
katG	Catalase-peroxidase	P13029	+1.93
acpP	Acyl carrier protein	P0A6A8	+1.89
pal	Peptidoglycan-associated lipoprotein	P0A912	+1.86
deoD	Purine nucleoside phosphorylase DeoD-type	P0ABP8	+1.82

cmk	Cytidylate kinase	P0A6I0	+1.80
eptC	Phosphoethanolamine transferase EptC	P0CB39	+1.79
yibT	Uncharacterized protein YibT	Q2M7R5	+1.79
glpQ	Glycerophosphodiester phosphodiesterase, periplasmic	P09394	+1.76
pdxJ	Pyridoxine 5'-phosphate synthase	P0A794	+1.76
maeB	NADP-dependent malic enzyme	P76558	+1.75
rplL	50S ribosomal protein L7/L12	P0A7K2	+1.71
glpD	Aerobic glycerol-3-phosphate dehydrogenase	P13035	+1.70
dapA	4-hydroxy-tetrahydrodipicolinate synthase	P0A6L2	+1.69
prlC	Oligopeptidase A	P27298	+1.68
cpdB	2',3'-cyclic-nucleotide 2'-phosphodiesterase/3'-nucleotidase	P08331	+1.68
ribE	6,7-dimethyl-8-ribityllumazine synthase	P61714	+1.65
atpF	ATP synthase subunit b	P0ABA0	+1.63
rpe	Ribulose-phosphate 3-epimerase	P0AG07	+1.61
hdhA	7alpha-hydroxysteroid dehydrogenase	P0AET8	+1.60
ridA	2-iminobutanoate/2-iminopropanoate deaminase	P0AF93	+1.59
gmhA	Phosphoheptose isomerase	P63224	+1.59
aspC	Aspartate aminotransferase	P00509	+1.57
trxA	Thioredoxin 1	P0AA25	+1.56
yjbJ	UPF0337 protein YjbJ	P68206	+1.53
acrA	Multidrug efflux pump subunit AcrA	P0AE06	+1.52
zapB	Cell division protein ZapB	P0AF36	+1.52
tpx	Thiol peroxidase	P0A862	+1.51
pgk	Phosphoglycerate kinase	P0A799	+1.51
ptsl	Phosphoenolpyruvate-protein phosphotransferase	P08839	+1.49
nusA	Transcription termination/antitermination protein NusA	P0AFF6	+1.49
pspA	Phage shock protein A	P0AFM6	+1.48
cydA	Cytochrome bd-I ubiquinol oxidase subunit 1	P0ABJ9	+1.47
gnd	6-phosphogluconate dehydrogenase, decarboxylating	P00350	+1.46
dnaK	Chaperone protein DnaK	P0A6Y8	+1.44
sdhB	Succinate dehydrogenase iron-sulfur subunit	P07014	+1.42
htpG	Chaperone protein HtpG	P0A6Z3	+1.42
ackA	Acetate kinase	P0A6A3	+1.42
ahpC	Alkyl hydroperoxide reductase C	P0AE08	+1.42
tsf	Elongation factor Ts	P0A6P1	+1.41
rpsA	30S ribosomal protein S1	P0AG67	+1.37
oppA	Periplasmic oligopeptide-binding protein	P23843	+1.34
ompA	Outer membrane protein A	P0A910	+1.31
aceF	Dihydrolipoyllysine-residue acetyltransferase component of pyruvate dehydrogenase complex	P06959	+1.30
groEL	Chaperonin GroEL	P0A6F5	+1.27
putA	Bifunctional protein PutA	P09546	+1.25
eno	Enolase	P0A6P9	+1.22

rplJ	50S ribosomal protein L10	P0A7J3	+1.21
rpsC	30S ribosomal protein S3	P0A7V3	-1.02
sucD	Succinate--CoA ligase [ADP-forming] subunit alpha	P0AGE9	-1.03
rplI	50S ribosomal protein L9	P0A7R1	-1.04
rplE	50S ribosomal protein L5	P62399	-1.05
gatD	Galactitol 1-phosphate 5-dehydrogenase	P0A9S3	-1.05
rplR	50S ribosomal protein L18	P0C018	-1.06
rho	Transcription termination factor Rho	P0AG30	-1.06
gapA	Glyceraldehyde-3-phosphate dehydrogenase A	P0A9B2	-1.06
raiA	Ribosome-associated inhibitor A	P0AD49	-1.06
rplM	50S ribosomal protein L13	P0AA10	-1.06
ptsH	Phosphocarrier protein HPr	P0AA04	-1.06
rpsL	30S ribosomal protein S12	P0A7S3	-1.07
rpsS	30S ribosomal protein S19	P0A7U3	-1.07
infC	Translation initiation factor IF-3	P0A707	-1.07
pta	Phosphate acetyltransferase	P0A9M8	-1.07
tnaA	Tryptophanase	P0A853	-1.07
pyrG	CTP synthase	P0A7E5	-1.08
rpmG	50S ribosomal protein L33	P0A7N9	-1.08
rplB	50S ribosomal protein L2	P60422	-1.08
rpmC	50S ribosomal protein L29	P0A7M6	-1.08
aceA	Isocitrate lyase	P0A9G6	-1.09
rpsE	30S ribosomal protein S5	P0A7W1	-1.10
ydcH	Uncharacterized protein YdcH	P0ACW6	-1.10
hns	DNA-binding protein H-NS	P0ACF8	-1.10
rpsR	30S ribosomal protein S18	P0A7T7	-1.10
rplX	50S ribosomal protein L24	P60624	-1.11
rplV	50S ribosomal protein L22	P61175	-1.11
rpsF	30S ribosomal protein S6	P02358	-1.11
hslU	ATP-dependent protease ATPase subunit HslU	P0A6H5	-1.11
rplF	50S ribosomal protein L6	P0AG55	-1.12
skp	Chaperone protein Skp	P0AEU7	-1.13
glyA	Serine hydroxymethyltransferase	P0A825	-1.13
rpsG	30S ribosomal protein S7	P02359	-1.13
dapD	2,3,4,5-tetrahydropyridine-2,6-dicarboxylate N-succinyltransferase	P0A9D8	-1.13
degP	Periplasmic serine endoprotease DegP	P0C0V0	-1.14
rplY	50S ribosomal protein L25	P68919	-1.14
rpsJ	30S ribosomal protein S10	P0A7R5	-1.14
rpsD	30S ribosomal protein S4	P0A7V8	-1.15
rpsT	30S ribosomal protein S20	P0A7U7	-1.15
rpsI	30S ribosomal protein S9	P0A7X3	-1.16
rpsH	30S ribosomal protein S8	P0A7W7	-1.17

rplW	50S ribosomal protein L23	P0ADZ0	-1.17
stpA	DNA-binding protein StpA	P0ACG1	-1.18
tufB	Elongation factor Tu 2	P0CE48	-1.18
tufA	Elongation factor Tu 1	P0CE47	-1.18
pykA	Pyruvate kinase II	P21599	-1.18
rplP	50S ribosomal protein L16	P0ADY7	-1.18
rpmB	50S ribosomal protein L28	P0A7M2	-1.19
cysK	Cysteine synthase A	P0ABK5	-1.19
rpoC	DNA-directed RNA polymerase subunit beta	P0A8T7	-1.20
glyS	Glycine--tRNA ligase beta subunit	P00961	-1.20
rpmI	50S ribosomal protein L35	P0A7Q1	-1.21
rpmH	50S ribosomal protein L34	P0A7P5	-1.24
eco	Ecotin	P23827	-1.24
glnH	Glutamine-binding periplasmic protein	P0AEQ3	-1.27
accC	Biotin carboxylase	P24182	-1.28
csrA	Carbon storage regulator	P69913	-1.29
clpA	ATP-dependent Clp protease ATP-binding subunit ClpA	P0ABH9	-1.30
rplS	50S ribosomal protein L19	P0A7K6	-1.30
hupA	DNA-binding protein HU-alpha	P0ACF0	-1.30
mreB	Cell shape-determining protein MreB	P0A9X4	-1.32
argG	Argininosuccinate synthase	P0A6E4	-1.35
ydgH	Protein YdgH	P76177	-1.35
ppiA	Peptidyl-prolyl cis-trans isomerase A	P0AFL3	-1.37
rplN	50S ribosomal protein L14	P0ADY3	-1.37
srmB	ATP-dependent RNA helicase SrmB	P21507	-1.39
crp	cAMP-activated global transcriptional regulator CRP	P0ACJ8	-1.46
rpmF	50S ribosomal protein L32	P0A7N4	-1.48
cra	Catabolite repressor/activator	P0ACP1	-1.53
ytfQ	Galactofuranose-binding protein YtfQ	P39325	-1.57
rnr	Ribonuclease R	P21499	-1.60
rpsM	30S ribosomal protein S13	P0A7S9	-1.61
asnA	Aspartate--ammonia ligase	P00963	-1.77
lepA	Elongation factor 4	P60785	-1.90
spy	Periplasmic chaperone Spy	P77754	-3.25
galS	HTH-type transcriptional regulator GalS	P25748	-3.60
dnaX	DNA polymerase III subunit tau	P06710	-4.33
ppk	Polyphosphate kinase	P0A7B1	-5.00
mglA	Galactose/methyl galactoside import ATP-binding protein MglA*	P0AAG8	-5.50
pliG	Inhibitor of g-type lysozyme	P76002	-6.00
hrpA	ATP-dependent RNA helicase HrpA	P43329	-12.00
mglB	D-galactose-binding periplasmic protein*	P0AEE5	-OLR
rnpA	Ribonuclease P protein component	P0A7Y8	-OLR

mntR	Transcriptional regulator MntR	P0A9F1	-OLR
